# Building consensus on the application of organoid-based drug sensitivity testing in cancer precision medicine and drug development

**DOI:** 10.7150/thno.96027

**Published:** 2024-05-27

**Authors:** Dongxi Xiang, Aina He, Rong Zhou, Yonggang Wang, Xiuying Xiao, Ting Gong, Wenyan Kang, Xiaolin Lin, Xiaochen Wang, PDO-based DST Consortium, Lianxin Liu, Ye-Guang Chen, Shaorong Gao, Yingbin Liu

**Affiliations:** 1 State Key Laboratory of Systems Medicine for Cancer, Shanghai Cancer Institute, Shanghai Jiaotong University School of Medicine, Shanghai 200232, PRC; 2 Department of Biliary-Pancreatic Surgery, Renji Hospital Affiliated to Shanghai Jiaotong University School of Medicine, Shanghai 200127, PRC; 3 Department of Oncology, Shanghai Jiaotong University Affiliated Sixth People's Hospital, Shanghai 200233 PRC; 4 Department of Oral and Maxillofacial-Head and Neck Oncology, Ninth People's Hospital, Shanghai Jiaotong University School of Medicine, Shanghai 200125, PRC; 5 National Center of Stomatology, National Clinical Research Center for Oral Disease, Shanghai 200011, PRC; 6 Department of Oncology, Ren Ji Hospital, Shanghai Jiaotong University School of Medicine, Shanghai 200127, PRC; 7 Department of Oncology, Tianjin Medical University General Hospital, Tianjin 300052, PRC; 8 Department of Neurology and Institute of Neurology, Ruijin Hospital Affiliated to Shanghai Jiaotong University School of Medicine, Shanghai 200025, PRC; 9 Department of Neurology, Ruijin Hospital Affiliated to Shanghai Jiaotong University School of Medicine (Boao Research Hospital), Hainan 571434, PRC; 10 Department of Surgical Oncology, Second Affiliated Hospital, Zhejiang University School of Medicine, No. 88, Jiefang Road, Hangzhou, Zhejiang 310009, PRC; 11 PDO-based DST Consortium; 12 Department of Hepatobiliary Surgery, The First Affiliated Hospital of USTC, Division of Life Sciences and Medicine, University of Science and Technology of China, Anhui 230001, PRC; 13 Anhui Province Key Laboratory of Hepatopancreatobiliary Surgery, Anhui Provincial Clinical Research Center for Hepatobiliary Diseases, Hefei, Anhui 230001, PRC; 14 The State Key Laboratory of Membrane Biology, Tsinghua-Peking Center for Life Sciences, School of Life Sciences, Tsinghua University, Beijing 100190, PRC; 15 The MOE Basic Research and Innovation Center for the Targeted Therapeutics of Solid Tumors, School of Basic Medicine, Jiangxi Medical College, Nanchang University, Nanchang 330047, China; 16 Translational Medical Center for Stem Cell Therapy & Institute for Regenerative Medicine, Shanghai East Hospital, School of Life Sciences and Technology, Tongji University, Shanghai 200120, PRC; 17 Frontier Science Center for Stem Cell Research, Tongji University, 1239 Siping Road, Shanghai 200092, PRC; 18 Shanghai Key Laboratory of Maternal-Fetal Medicine, Clinical and Translational Research Center of Shanghai First Maternity and Infant Hospital, School of Life Sciences and Technology, Tongji University, Shanghai 200092, PRC

**Keywords:** Organoid, Patient-derived organoids (PDOs), Precision medicine, Drug sensitivity testing (DST), Expert consensus

## Abstract

Patient-derived organoids (PDOs) have emerged as a promising platform for clinical and translational studies. A strong correlation exists between clinical outcomes and the use of PDOs to predict the efficacy of chemotherapy and/or radiotherapy. To standardize interpretation and enhance scientific communication in the field of cancer precision medicine, we revisit the concept of PDO-based drug sensitivity testing (DST). We present an expert consensus-driven approach for medication selection aimed at predicting patient responses. To further standardize PDO-based DST, we propose guidelines for clarification and characterization. Additionally, we identify several major challenges in clinical prediction when utilizing PDOs.

## Introduction

*Nature Methods* identified organoids as one of the top ten annual technologies in 2017 1. Patient-derived organoid (PDO) has become an attractive research platform for various cancer types, including colon 2, stomach 3, breast 4, prostate 5, liver 6, pancreas 7, neuroendocrine 8, glioblastoma 9, lung 10 and pediatric kidney cancers 11, etc. These organoid models are suitable for translational research, the development of new medications, and personalized care. Based on preclinical efficacy results obtained from the organoid-on-a-chip model, the US FDA approved *Sutimlimab* to commence clinical trials for the first time in July 2022 without the requirement for *in vivo* supporting data 12. This marks a significant milestone in the utilization of organoids for drug development.

According to the National Cancer Center of China's 2024 report 13, 14, the estimated number of new cancer cases reached 4.82 million in 2022 (*vs.* 4.06 million in the 2022 report). Remarkably, approximately 2.57 million cancer-related deaths occurred in China. As the incidence and mortality rate of cancer patients continue to escalate, it is imperative to develop precise preclinical models for tumor detection and treatment. In this article, we mainly concentrated on PDOs generated from solid tumors. Up to now, the techniques for culturing organoids of hematological malignancies remain immature, with only a few reports in the literature 15-17. This consensus paper outlines eight potential applications of PDOs in the fields of cancer precision medicine and drug discovery.

## 1. Tumor organoid definition

**Figure 1:**
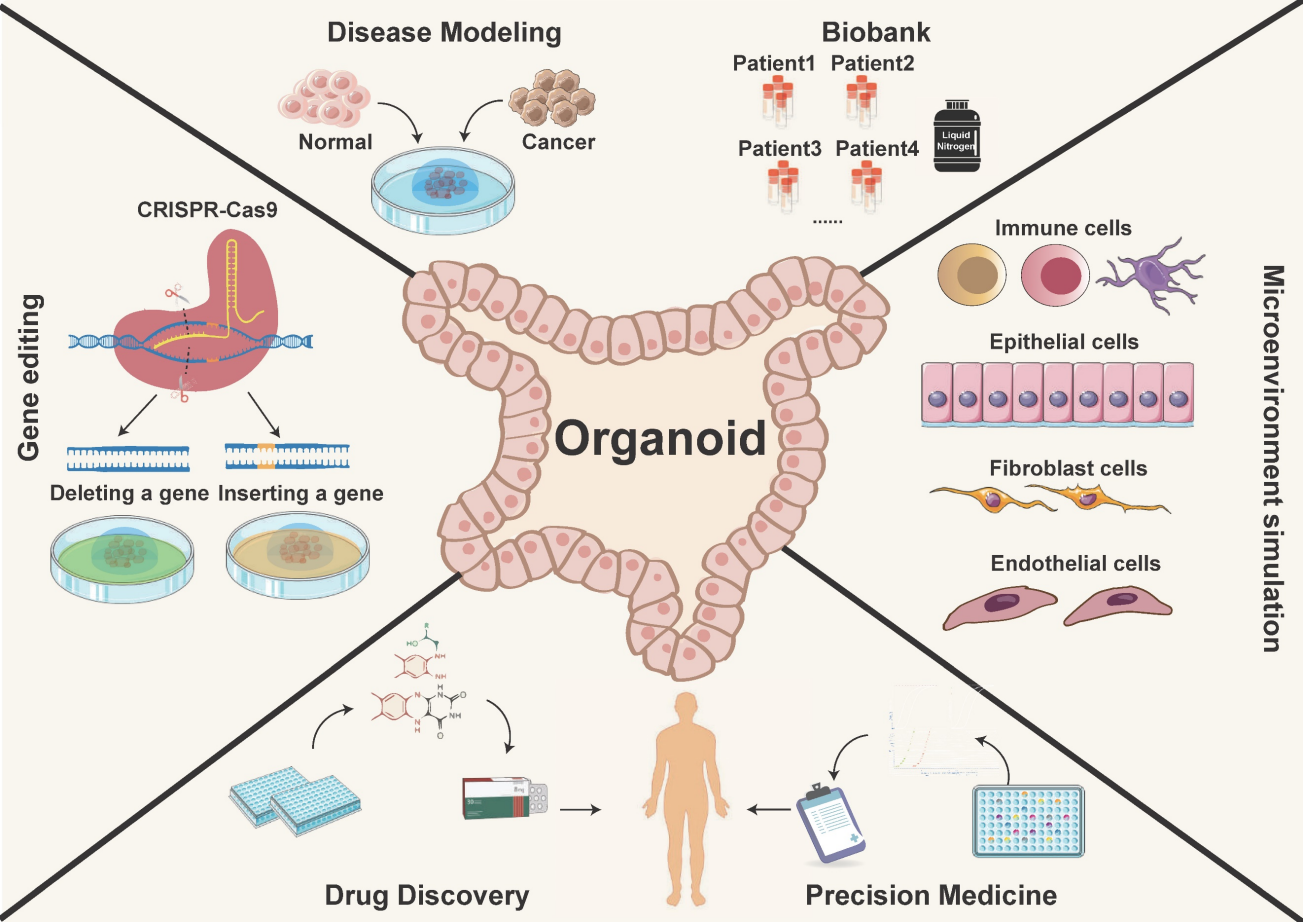
** The application of organoid models.** PDOs recapitulate the physiological features and function, providing a more authentic and effective technical platform. They have potential applications in various research areas such as constructing disease models, biological sample repositories, gene therapy, drug discovery, and precision medicine.

Tumor organoids are increasingly utilized in cancer research and personalized medicine. They are *in vitro* culture models with a three-dimensional (3D) structure and are self-differentiated from cells with stem cell characteristics. Tumor organoids typically allow for long-term expansion and recapitulate the intricate cellular heterogeneity and spatial architecture of the original tumor. They also mimic the functions and biological behaviors of the tissue origins, while retaining pathohistological features and several genetic traits, including mutation and amplification of driving genes 2, 4, 6, 8, 9, 18. PDOs are miniature, simplified versions of organs or tissues that can be grown in a laboratory setting and form a valuable platform for tumor research and drug discovery for the reasons outlined below (Figure 1) 19-21:

1) Constructing an *in vitro* model that precisely recapitulates the origin tumor 22. PDOs were generated by collecting tumor tissues from various cancer origins, including primary tumors, circulating tumor cells, and metastatic lesions 23. The tissue was then pre-processed under the action of mechanical and/or enzymatic digestion. Tumor cells were ultimately facilitated in 3D matrices to form cancerous organoids 16. These organoids feature a diverse array of cells, each with its unique role in the complex tapestry of tumor development and progression.

2) Establishing a living organoid biobank from tumor species 7. PDOs can be expanded for prolonged periods, allowing cryopreservation and recovery 24. Freezing the organoid when it is highly proliferative improves its recovery. PDOs are placed in a frozen vial containing freezing solution and left on ice for 30 minutes, then stored overnight at -80°C before transferring to liquid nitrogen for long-term storage. For organoid replating, thaw PDOs quickly at 37°C and place them in petri dishes for further manipulation 25. Inappropriate freezing/thawing single cells or large and differentiated organoids may result in poor recovery, morphological changes or potential impact on drug screening outcomes 26. Therefore, the principle of freezing/thawing should be followed, similar to cell lines, and PDOs must revive within 1-2 weeks to regain their expansion capacity after recovery.

3) *In vitro* tumor modeling allows for the manipulation of variables such as nutrient availability, oxygen supply, and drug exposure to investigate their impact on tumor cell behavior. These models replicate the structure and microenvironment of *in vivo* tumors, offering a standardized and realistic platform for studying phenomena like tumor growth, invasion, and therapeutic response.

4) Predict clinical responses of patients with various cancer types 27-29, including colorectal 2, 30, 31, breast 4, 32, 33, lung 34, 35, ovarian 36, urothelial 37, gastrointestinal 38 and pancreatic 23, 39 cancers. PDOs advance precision cancer medicine by enabling prospective therapy assessment. Researchers generate PDOs to screen therapeutic options that are potentially most effective. This personalized approach offers the potential for improving patient outcomes by optimizing treatment regimens and minimizing unnecessary side effects.

5) Develop genetically modified tumor organoid models 40, 41 and identify the development mechanisms of tumor gene mutations 42, 43. By applying CRISPR/Cas9 gene editing for tumor-driven genes, PDOs can be used to study tumor biology, investigate the mechanisms of drug resistance, and develop new therapeutic approaches for cancer treatment.

In summary, PDOs represent the cutting-edge intersection of biology, medicine, and technology. Their usages enable researchers to dissect the complexities of cancer, unravel its secrets, and develop personalized medicine (Figure 1). With their ability to faithfully recapitulate the cellular and molecular intricacies of tumors, PDOs serve as a testament to human ingenuity and an indispensable asset in the ongoing fight against cancer.

## 2. The role of PDOs in drug discovery

**Figure 2:**
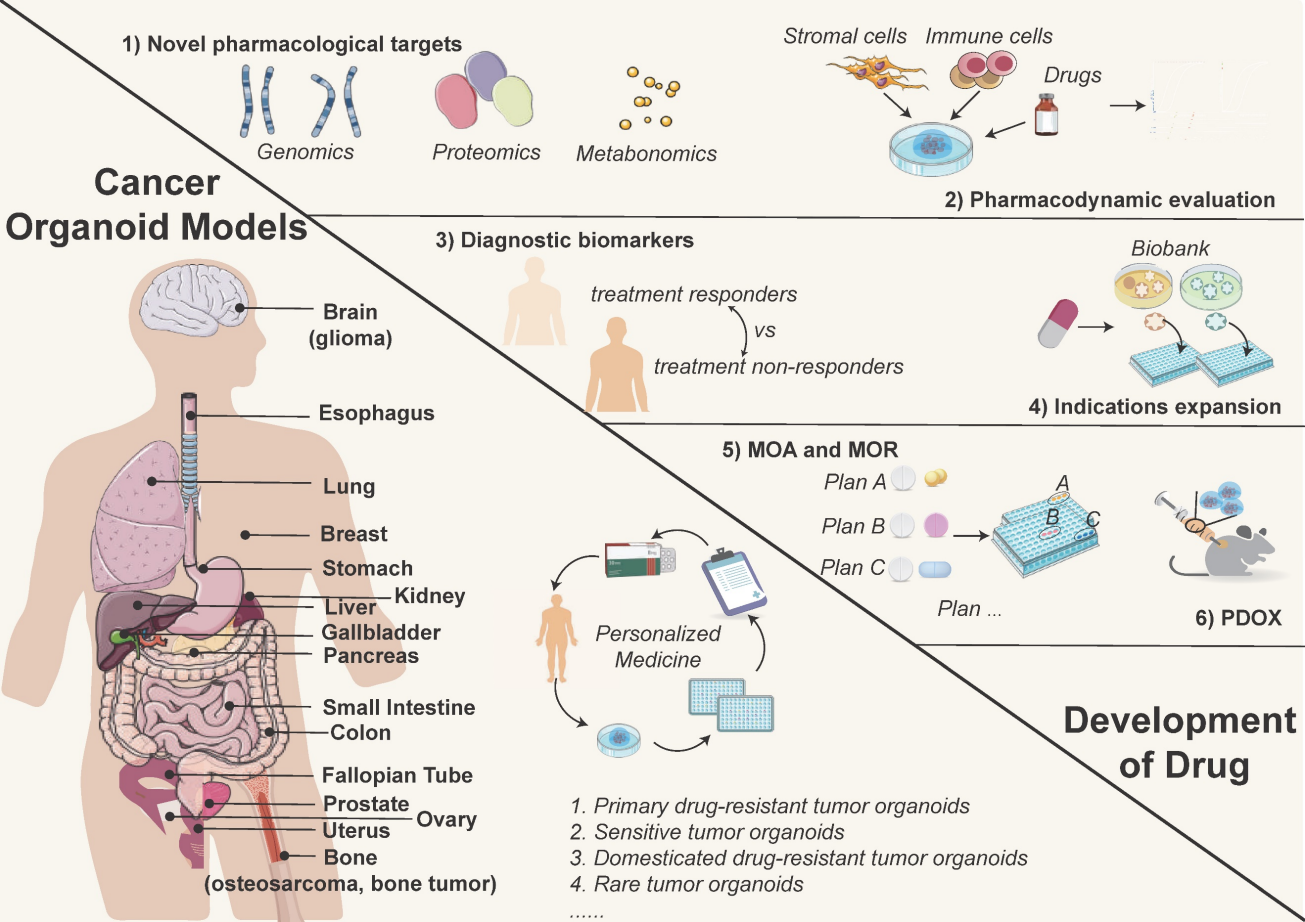
** Cancer organoid models used in precision medicine (left) and the opportunities for drug discovery (right).** Tumor organoids simulate the biological characteristics of tissues-of-origin, providing matched personalized treatment strategies. Currently reported tumor organoid models include but are not limited to esophageal, lung, breast, gastric, renal, colorectal, and liver cancers, *etc*. (left image). Meanwhile, tumor organoids are effective preclinical models for drug development, which can be used to discover novel drug targets, test drug dosages, explore diagnostic biomarkers, repurpose existing drugs, and conduct PDO-xenograft (PDOX) model, *etc*. (right image).

In preclinical research, PDOs serve as potent surrogates for understanding diseases, including cancer and various organ-specific disorders, and exploring new treatment options (Figure 2). By faithfully recapitulating the complex cellular architecture and functionality of the original tissue, PDOs provide a remarkable platform for probing disease mechanisms, elucidating molecular pathways, and exploring therapeutic interventions. With the generation of organoids from diverse tissue sources, they can be utilized for multi-omics and gene editing research, taking on an increasingly pivotal role in preclinical drug development, especially target finding (Figure 2).

***1) Identification and validation of novel pharmacological targets.*** A cancer cell line is a typical *in vitro* model for discovering novel therapeutic targets. However, two-dimensional cell lines are incapable of mimicking the essential 3D cell composition and heterogeneity of *in vivo* tumors, resulting in a success rate of less than 1% in novel drug development 28, 44. Organoids retain the unique genomic alterations, molecular signatures, and heterogeneity present in the original tissue, making them invaluable for studying disease progression and identifying potential therapeutic targets. Multi-omics investigations, including genomics 45, proteomics 46, 47, and metabolomics 48, further enhance the reliability of applying PDOs for discovering novel therapeutic targets. Organoids can be treated using technologies like CRISPR/Cas9, RNAi, or gene overexpression etc. for therapeutic target validation 49. Additionally, co-culture systems involving organoids and mesenchymal/immune cells offer great opportunities for studying therapeutic targets other than tumor cells 50.

Normal tissue-derived organoids play valuable roles in novel pharmacological discovery. Human induced pluripotent stem cell-derived motor neurons (hiPSC-MN) provide insights into neurobiological mechanisms in motor neuron diseases such as spinal muscular atrophy and spinal/bulbar muscular atrophy 51. These hiPSC-derived 3D organoids facilitate disease pathogenesis study and therapeutic discovery and validation. Additionally, in lung epithelial repair, cigarette smoke extract-exposed organoids exhibit defective lung epithelial progenitors, restored by prostaglandin E2 and prostacyclin 52. Normal PDOs used for cancer drug discovery normally allow for therapy side effect monitoring 53.

***2) Drug screening.*** Tumor organoids retain patients' therapeutic targets and preserve the genetic and phenotypic variability of malignancies 54, 55. These can be available for assessing the efficacy of specific medications or pharmacological combinations. Co-culture systems also allow for the inclusion of non-tumor cell types like stromal and immune cells, enabling the evaluation of additional treatment types 56.

***3) Discovery of diagnostic companion biomarkers.*** Biomarkers can be identified by predicting the therapeutic efficacy of targeted medications and comparing variations in multi-omics characteristics between treatment responders and non-responders 57. Organoids serve as a crucial platform for discerning potential diagnostic biomarkers, which are subsequently validated via analysis of clinical samples. For instance, extracellular vesicle protein and PTP4A1 have been identified as diagnostic biomarkers for pancreatic ductal adenocarcinoma and mucoepidermoid carcinoma, respectively 58, 59.

***4) Expanding*** ***indications for medications*****.** A comprehensive and diverse cancer organoid biobank can be utilized to test pharmaceuticals still in clinical trials to expand their treatment indications in addition to medicines under development 7. The cancer organoid biobank combined with high-throughput drug screening can reveal sensitivity to unexpected drugs 3. For example, the human gastric cancer organoid biobank showed sensitivity to Napabucasin, Abemaciclib, and VE-822 3.

***5) Examining the mechanism of action (MOA) and mechanism of resistance (MOR) of drugs*.** As an *in vitro* model with greater therapeutic relevance than cell lines, tumor organoids can be a valuable tool for analyzing the MOA and MOR of therapeutics. Drug resistance is unavoidable in cancer therapy, and organoids can be utilized to investigate drug resistance mechanisms comprehensively and efficiently. Primary drug-resistant tumor organoids, sensitive tumor organoids, and domesticated drug-resistant tumor organoids are all high-quality resources for studying MOR 60. A multiplex organoid-based drug response assay was conducted in pancreatic ductal adenocarcinoma, utilizing the area under the curve (AUC) to identify predictors of drug sensitivity associated with the MOA 61. Cong *et al.* studied how colon cancer cells evade drug action by enhancing metabolism, identifying specific metabolites involved in drug resistance 62. The study of seven epithelial ovarian cancer PDOs accurately reflected the clinical response of patients to platinum-based chemotherapy 63. Similarly, PDOs derived from colorectal cancer peritoneal metastases predicted responses to cytoreductive surgery followed by hyperthermic intraperitoneal chemotherapy 30. Furthermore, an ovarian cancer PDO demonstrated its suitability for drug resistance testing and screening in the context of personalized medicine 64. Molecular and therapeutic profiling of pancreatic cancer PDOs demonstrated accuracy in predicting drug resistance, further advancing personalized medicine 23.

***6) Pharmacokinetics and pharmacodynamics in vivo studies*.** The organoid model has been utilized for pharmacokinetic/pharmacodynamic (PK/PD) testing to evaluate the efficacy and safety of pharmaceuticals. It is also integrated with high-throughput technology for efficient compound screening. Han *et al.* established lung and colon organoid models using humanized pluripotent stem cells (hPSCs) and conducted high-throughput screening of FDA-approved drugs. Three small molecule drugs were identified as potent inhibitors of SARS-CoV-2 cell entry, indicating their potential as candidates for COVID-19 treatment 65. The Patient-Derived Tumor Xenograft Organoid Model (PDXO) can be generated from both Patient-Derived Tumor Xenografts (PDX) and the PDO Xenograft Model (PDOX). PDXOs are instrumental in evaluating the efficacy of lead compounds and other clinical candidates. These models enable the verification of candidates with the most promising pharmacological outcomes and facilitate the analysis of their pharmacokinetics, optimal dosage, and administration schemes 66. PDO has a higher success rate of formation compared to PDX, significantly increasing the likelihood of creating *in vitro* and *in vivo* model pairings for the same original tumor material 38.

***7) Organoid-based prediction of radiation response*.** The organoid model offers new insights into tumor radiation sensitivity. Martin *et al.* examined how the colorectal and intestine organoids responded to ionizing radiation 67. Researchers assessed the *in vitro* radiation-mitigating effects of SIRT1 inhibitors on organoid survival rates and size changes post-irradiation 68. These studies using functional organoid models may inform medical strategies against radiation-induced damage.

The role of PDOs in preclinical research is transformative (Figure 2). These engineered 3D models faithfully mimic the characteristics of patient tissues, serving as potent surrogates for investigating disease mechanisms, testing novel therapeutics, and advancing personalized medicine approaches. PDOs stand as a promising frontier in medical research, offering hope for better treatments, improved patient outcomes, and ultimately, a more promising future in combating diseases.

**Expert consensus 1:** PDOs offer extensive application prospects in preclinical research, and can be utilized for the discovery and validation of novel therapeutic targets, pharmacodynamic evaluation, detection of simultaneous diagnostic biomarkers, expansion of pharmacological indications, exploration of drug action and resistance mechanisms, and *in vivo* pharmacokinetics, etc.

## 3. Tumor organoid models for preclinical studies

Tumor organoid models are particularly effective in their ability to replicate the tumor microenvironment. Cells interact with each other as well as with the surrounding extracellular matrix (ECM), facilitating the study of key interactions that influence tumor behavior, immune responses, and drug resistance. This physiological relevance offers a more comprehensive understanding of tumor biology and enables the exploration of new therapeutic targets and treatment approaches. Tumor organoids can fill the gap where preclinical models for numerous diseases are lacking (Figure 2):

***1) Tumor organoids are derived from* rare types of cancer.** Many malignancies with particularly low incidence rates, such as glioma 69, 70, bone tumor, osteosarcoma 71, pediatric tumors, lack reliable preclinical research models. Tumor organoids stand as groundbreaking solutions to this critical gap in research.

***2) Tumor organoids containing novel therapeutic targets are lacking in tumor cell lines*.** Existing tumor cell lines are inadequate in recapitulating complex spatial structure, and microenvironment of tumors, which results in a lack of novel therapeutic targets. PDOs can facilitate drug development by identifying these targets 72. Driehuis *et al.* reported that PDOs from head and neck cancer patients recapitulate EGFR expression levels, validating their relevance superior to cell lines 73. The absence of neuroendocrine neoplasms (NEN) pre-clinical models poses challenges. Dayton *et al.* established PDOs from NEN patients, maintaining the gene expression pattern, tumor heterogeneity and evolutionary process *in vitro* 74. Besides, vascularized organoids provide an appreciated platform for testing anti-angiogenic drugs targeting VEGF/VEGFR 75. For immunotherapy, co-culture of tumor organoids and immune cells preserves essential tumor-related antigens crucial for immune-related therapies, such as immune checkpoint inhibitors (PD-1/PD-L1) 76, immune cell therapies (TILs, CAR-T), pattern recognition receptors (PRRs), *etc.* 77.

***3) Drug-resistant tumor organoids***. Antitumor therapeutics often eventually lead to drug resistance, causing treatment failure. Drug resistance-based tumor organoid models can facilitate the study of MOR 60, 78.

Normal organoids are vital in drug development for both tumor and non-tumor conditions. They contribute significantly to predicting adverse reactions, drug metabolism, and toxicological evaluation 79-81. For instance, liver organoids, mimicking normal liver cell composition and function provides a physiologically relevant environment for *in vivo* chemical processing, making them valuable for drug validation and toxicity assessment 79-81.

**Expert consensus 2:** Preclinical studies on pharmaceuticals largely depend on robust models. PDOs are well-suited for studying rare malignancies, tumors for which no existing models exist, and drug-resistant species.

## 4. Organoids in cancer clinical practice

The clinical efficacy of targeted and immunotherapeutic treatments often falls short of expectations. Next-generation sequencing for identifying genetic alterations has shown limited value in guiding patients to most treatments 82, 83. Precision medicine, based on the unique pharmacodynamic phenotypes of each patient, remains imprecisely defined. Numerous cancer PDOs exhibit comparable drug sensitivity to patients in clinical trials, including metastatic gastrointestinal and colorectal cancers 38, 84, 85, etc. (Table 1). By subjecting organoids to different treatment regimens, researchers can assess both response and potential side effects before initiating clinical trials. This preclinical screening using organoids optimizes the drug development process, increasing the likelihood of success in subsequent clinical phases while minimizing the risks and costs associated with traditional approaches.

Organoids facilitate the exploration of precision medicine in clinical trials. By establishing patient-specific organoid models, researchers can evaluate how these organoids respond to different treatment options, thereby identifying the most effective therapeutic regimen for individual patients. Application of organoid models in clinical trials must be meticulously planned and executed; as of September 3, 2023, 159 organoid-related projects had been registered on ClinicalTrial.Gov for cancer research, including lung 26, breast 4, pancreatic 86, head and neck 87, liver 88, ovarian 89, kidney 90, prostate cancers 91, etc. (Table 1). The first category of these clinical trials is non-interventional retrospective analyses or observational contemporaneous (co-clinical) studies (now 62/149) based on organoid DST. The second group (82/149) is prospective, interventional research, which provides more reliable data for customized treatments based on organoid DST. Key application scenarios include:

***1) Neoadjuvant treatment***: For patients with locally advanced cancer, prospective and interventional clinical studies are conducted to screen tumor therapeutic options and predict potential beneficiaries of neoadjuvant therapy.

***2) Salvage therapy***: For patients at high risk of recurrence or those who have already experienced recurrence, prospective and interventional clinical studies aim to identify potential and additional treatment beneficiaries.

***3) Palliative care***: Prospective and interventional clinical studies are conducted to assess potential beneficiaries of precise treatment for malignancies with recurrence, metastasis, and failed first- or second-line therapies in patients with no surgical indications 92.

**Expert consensus 3:** Clinical trials have shown that the results of organoid DST align closely with the actual clinical outcomes of patients. Integrating organoid DST into clinical research can potentially serve as a predictive biomarker of clinical treatment response.

## 5. Has the time come for clinical laboratory-developed test (LDT) exploration of organoid DST?

LDT mode is an *in vitro* diagnostic item that has not yet gained product registration and is developed, verified, and applied in the laboratory. Accelerating the development of LDT programs to support precision medicine is a growing trend 93, 94. Under the supervision of licensed physicians, accredited medical institutions can produce *in vitro* diagnostic reagents tailored to the clinical needs of their units. The exploration of using organoid DST in clinical laboratory-developed tests (LDTs) is an area of ongoing research and development. Organoid DST involves testing patient-derived organoids against various drugs or treatment regimens to measure their response and sensitivity.

**Figure 3:**
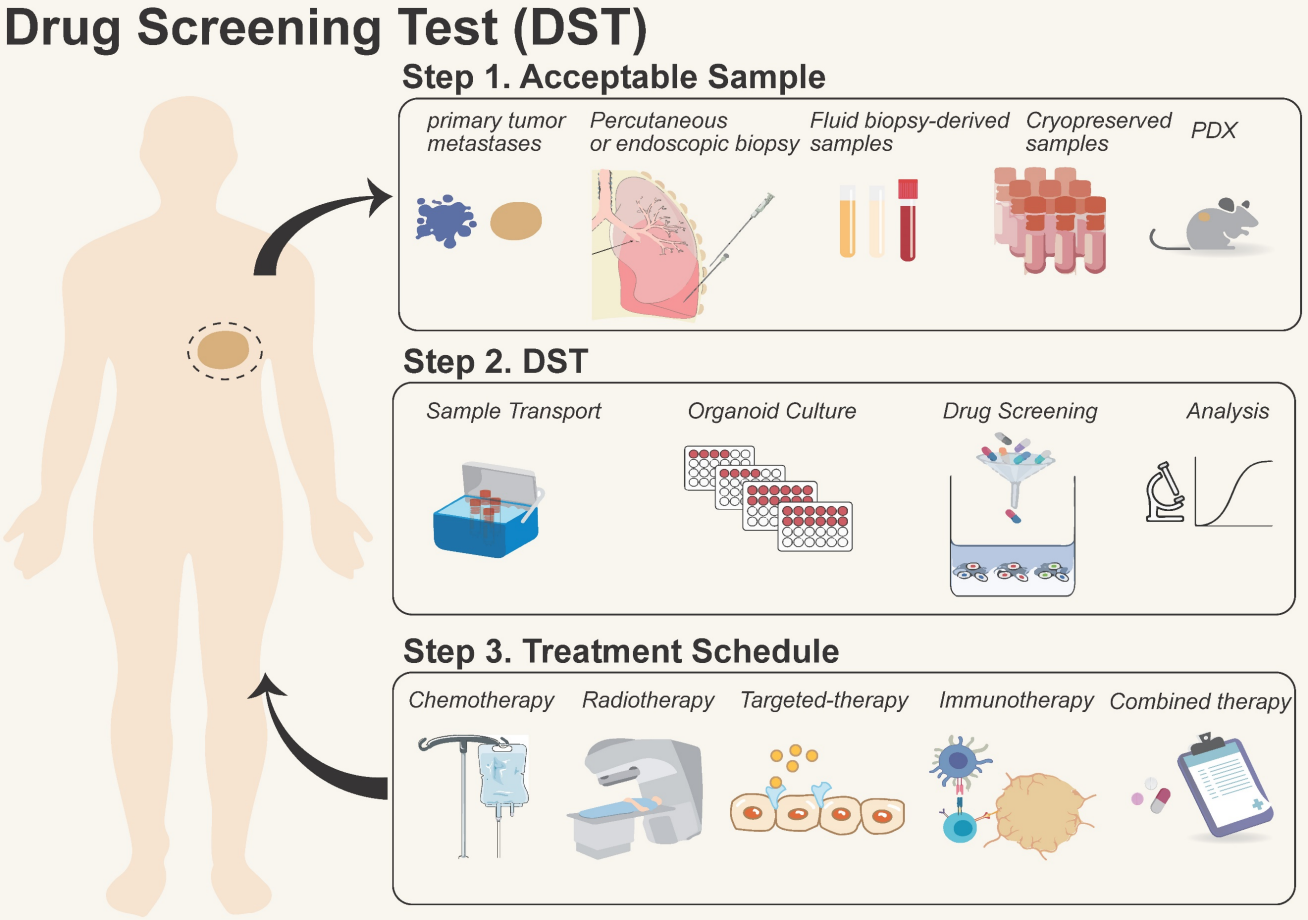
** Process of organoid models for drug screening test (DST).** They are summarized in three steps: 1) Sample acquisition: primary/metastatic tumors, biopsies, liquids, and patient-derived xenograft (PDX) samples, *etc*.; 2) Drug screening: including sample transportation, organoid formation, drug screening, and data analysis; 3) Treatment in the clinic: providing therapeutic options for patients with chemotherapy, radiotherapy, targeted therapy, immunotherapy, and combinational therapy, etc.

Organoid models for DST have been carried out *in vitro* for a wide range of solid cancers, including breast 4, bladder 95, gastric 3, 96, and rectal cancers 97, etc. These models have confirmed the consistency between laboratory data and clinical outcomes, which provides a practical approach to examining the mechanisms of sensitivity and resistance 98, 99. While further research is required to determine whether organoid DST improves the disease-free survival and overall survival of patients 100, 101, the incorporation of organoid DST platforms provided by the industry into hospital-based LDT programs is encouraged for patient benefit. By incorporating organoid DST into clinical LDTs, healthcare providers may be able to tailor treatment decisions based on the unique characteristics of a patient's tumor, ultimately improving treatment outcomes (Figure 3). The following precautions merit consideration when incorporating organoid DST into clinical LDT:

1) The implementation of organoid DST in clinical LDTs requires rigorous validation, standardization, and regulatory approval to ensure its accuracy, reproducibility, and clinical utility. This involves navigating various regulatory frameworks and meeting stringent criteria to ensure patient safety and the reliability of test results.

2) The adoption of organoid DST in clinical LDTs may also be influenced by factors such as healthcare system regulations, reimbursement policies, and the availability of resources and expertise in conducting such tests.

3) It is important to note that the field of LDTs is continually evolving, and the decision to explore organoid DST in clinical LDTs would depend on multiple factors, including scientific evidence, regulatory considerations, and the needs and priorities of the healthcare community.

4) For the most accurate and up-to-date information on the status and potential future developments in organoid DST and its incorporation into clinical LDTs, it is advisable to consult scientific literature, regulatory authorities, and healthcare experts specialized in the field.

Motivated by the current progress in clinical exploration of organoid DST, the transition from laboratory development to commercialization is imminent:

1) Laboratory development: LDTs were developed and implemented within individual laboratories to address specific needs unmet by available tests. As of May 2023, the Centers for Medicare & Medicaid Services report over 277,251 CLIA registered laboratories in the U.S., with fewer than 6% of high-complexity laboratories capable of conducting LDTs.

2) Regulatory oversight and quality control: Concerns about the quality and consistency of LDTs have led regulatory bodies such as the U.S. FDA to consider enhanced oversight. In October 2023, the FDA proposed ending enforcement discretion for LDTs, aiming for more stringent regulation.

3) Transition to commercialization: the shift from LDTs to commercialized tests involves:

A. Validation and verification: These processes precede commercialization, ensuring accuracy and reproducibility across diverse populations and conditions.

B. Regulatory approval: Commercial tests undergo regulatory scrutiny, including assessment of analytical and clinical performance, and compliance with safety and efficacy standards.

C. Manufacturing scale-up: Upon regulatory approval, manufacturers scale up production, optimizing processes and quality control to meet market demand.

D. Distribution and marketing: Commercialized tests are distributed through various channels, with targeted marketing strategies to promote their benefits and expand market reach.

**Expert Consensus 4:** Organoid DST has reached clinical service; accredited institutions are encouraged to refine their LDT protocols.

## 6. Which patients would benefit from organoid DST?

In the clinical practice of cancer treatment, the identification and judicious application of molecular markers are essential. However, fewer than 7% of patients can benefit from precision medicine through next gene sequencing 83. Vlachogiannis reported in 2018 that organoid DST achieved a negative and positive predictive value of 100% and 88%, respectively, in advanced gastrointestinal cancer, paving the way for clinical application of PDO 38. Organoid DST can provide valuable insights into a patient's response to specific drugs or treatment regimens, thereby guiding treatment decisions and improving patient outcomes. It's reported that pancreatic cancer organoid DST could predict the curative effect and degree of gemcitabine efficacy 102. Yao *et al.* established 18 rectal cancer organoids for DST, achieving accuracies, sensitivity, and specificity of 84.43%, 78.01%, and 91.97%, respectively 103. Wang *et al.* assessed the treatment response of colorectal PDOs, the DST results showed the value of sensitivity (63.33%), specificity (94.12%), accuracy (79.69%) and positive predictive rate (90.48%) 31. Here are some scenarios where organoid DST could be beneficial:

***1) Patients with solid tumor.*** Current organoid technology is primarily designed for epithelial cells, making it suitable for most solid tumors originating from the epithelium (*i.e.*, colorectal 2, 30, 31, breast 4, 32, 33, lung 34, 35, ovarian 36, and pancreatic 39 cancers). Hematological cancers, unlike solid tumors, originate in the bone marrow and affect the growth of white blood cells. Tumor cells exist in fast-moving blood, dispersing throughout the body. Current reports support the *in vitro* organoid culture of hematoma and glioblastoma 104, 105.

***2) Patients receiving neoadjuvant chemotherapy*.** Neoadjuvant chemotherapy refers to systemic chemotherapy administered before local treatment (such as surgery or radiotherapy). This can reduce tumor size, eliminate micrometastases at the maximal level, and downgrade the disease, thereby facilitating subsequent treatment. A biopsy obtained through puncture is exposed to organoid culture and DST before administering neoadjuvant chemotherapy. As a prescription, the most effective treatment is then picked 106-108.

***3) Patients considering targeted therapies*** 32, 109, 110. Organoid DST can help predict the response to targeted treatments for patients with specific genetic mutations or alterations. This information can guide treatment decisions and prevent unnecessary exposure to ineffective drugs.

***4) Patients with rare or hard-to-treat cancers*** 110***.*** Organoid DST can help identify potential targeted therapies or novel drug combinations that may be more effective for rare or hard-to-treat cancer types. These types of cancer may have limited treatment options or suboptimal response rates to standard therapies.

***5) Patients whose initial treatment was unsuccessful*.** Patients who receive ineffective first-line therapy may benefit from organoid DST on primary or recurrent tumor biopsies, depending on the various therapeutic combinations used. Evaluation can improve the prognosis of patients who benefit from second-line treatment or unconventional therapy 111.

***6) Patients with recurrent or metastatic disease*** 30, 112***.*** Organoid DST can be useful for patients with recurrent or metastatic diseases, where treatment decisions become more complex. By testing the sensitivity of organoids derived from metastatic sites, clinicians can gain insights into the best treatment options and potentially identify therapies that are effective against specific metastatic lesions.

***7) Patients with tumors resistant to radiotherapy*** 39, 97, 113, 114***.*** Radiotherapy is the initial treatment for nasopharyngeal, cervical, and skin cancer. When radiation resistance occurs, organoid models provide patients with a selection of potential life-expending drugs. By predicting the sensitivity of the primary tumor, organoids also improve the achievement ratio of radiation for patients who undergo selective preoperative radiation therapy.

***8) Patients with advanced cancers*** 31, 112**.** There are no surgical indications for patients with advanced stages or metastases; chemo- and cell- therapies have become the standard of care. Before treatment, a biopsy and organoid DST could be performed, enabling the selection of targeted approaches. If drug resistance develops after treatment, a DST of post-resistance organoids can be performed 103.

***9) Patients with limited treatment options*** 112***.*** Organoid DST is valuable for patients with limited treatment options due to factors such as prior treatment failures, drug resistance, or specific tumor characteristics. By assessing the drug sensitivity of organoids, clinicians can identify alternative treatment strategies and potentially repurpose existing drugs for personalized treatment approaches.

The clinical utility and applicability of organoid DST are still being investigated and refined. The decision to use organoid DST for a specific patient would depend on several factors, including the type of disease, treatment history, testing resources, and clinical judgment.

**Expert Consensus 5:** Both organoid DST and next-generation sequencing belong to the field of precision medicine. However, organoid DST has more advanced application scenarios, such as neoadjuvant and/or palliative chemotherapy, ineffective first-line treatment, advanced and rare cancer, and metastatic tumors, etc., and can be implemented throughout the entirety of cancer treatment.

## 7. What types of samples are acceptable for organoid culture?

Organoid culture can be established using various types of samples depending on the specific research or clinical goals. The suitability of different sample types for organoid culture can vary based on factors such as the organ of interest, accessibility, and preservation of tissue integrity. The collection of high-quality samples is essential for the organoid establishment. After surgically removing a sample, it must be stored in a preservative solution at a specific temperature (e.g., 4°C) and transported to the laboratory within 48 hours 115. The success rate is higher when a greater quantity of tumor cells is collected, while avoiding necrotic, damaged, or fibrotic tissues. The sampling requirements for various source tissues are refined as follows.

***1) Samples from recent surgical resection*.** Immediately following surgery, organoid seeding samples should be collected, including the primary tumor and metastases (should be preserved in two to three peanut-grain-sized fragments, > 50 mg recommended). These specimens had the highest success rate for organoid culture and the most reliable responses in drug testing 9, 116. This is particularly relevant for studying organ-specific diseases or investigating the response of specific tissues to drugs or treatments.

***2) Percutaneous or endoscopic biopsy*.** Tumor biopsies obtained through minimally invasive procedures or surgical resections are frequently used for organoid culture. Organoid culture can be performed with 1-2 puncture sutures from primary and metastatic tumors, or 1-2 tissue fragments clamped under a gastrointestinal endoscope. The challenge with these samples lies in determining whether they are sufficient for DST at the puncture site, which is crucial for evaluating the overall efficacy of the medication 95, 117. The length of puncture specimens should reach one centimeter, preparing two to three puncture samples. For endoscopic biopsies, 1-2 tissue fragments are typically clamped under the guidance of an endoscope for further analysis.

***3) Fluid biopsy-derived samples*.** Blood (> 5 ml), urine (> 5 ml), pleural (> 2 ml), ascites (> 2 ml), peritoneal (> 2 ml), pericardial effusions and other liquid biopsies are essential for clinical diagnosis and translational research. These fluid body specimens have been utilized effectively for organoid models 118, 119. Fluid-based organoids can provide insights into the behavior of cancer cells in a systemic context and help monitor disease progression or treatment response (Table 2).

***4) Cryopreserved samples*.** For subsequent organoid culture, it is also possible to use shredded frozen tissue or frozen pre-digested single cells that have been stored at -80°C or liquid nitrogen. However, cryopreserved tissue has a lower success rate in establishing organoids and requires a longer expansion period 86.

***5) Patient-derived xenografts (PDX).*** PDX models involve transplanting patient tumor tissue directly into immunocompromised mice. These tumor xenografts can be subsequently used to generate organoids, allowing for the propagation of patient-specific tumor characteristics in a laboratory setting.

**Expert Consensus 6:** For organoid DST, small surgical biopsies obtained through puncture or gastrointestinal endoscopy can be used for cancer tissues. The success of organoid culture depends on factors such as the viability and quality of cells in the starting tissue sample, as well as the specific protocols and culture conditions used for organoid generation. Researchers and clinicians need to consider these factors when selecting the most appropriate sample type for their study or clinical application.

## 8. Which patient treatment approaches can be utilized via organoid DST?

***1) Chemotherapy*.** Chemotherapy is currently the predominant therapeutic modality for solid tumors and the main direction of organoid DST, which comprises monotherapy and combined treatment. The correlation between organoid DST and clinical response has been extensively reported 3, 24, 38, 97.

***2) Radiotherapy*.** Radiotherapy, like chemotherapy, has attracted much interest in organoid DST, including single-agent chemotherapy paired with radiotherapy 39, 97, 113, 114.

***3) Targeted therapy*** 32, 109, 110**.** Targeted therapy is underutilized in most solid tumors, excluding lung and breast malignancies, for which targeted therapy is relatively well-established. For clinical diagnosis and therapy recommendations, organoid DST, histological labeling, and large-scale, high-throughput genetic mutation screening may become commonplace 48, 120, 121.

***4) Immunotherapy*** 77, 122, 123**.** Immunotherapy is promising for cancer treatment. However, its clinical efficacy prediction using organoid DST requires additional research:

*A. How to maintain the immune microenvironment in vitro*. Organoids can either be co-cultured with immune cells and elements of the innate immune microenvironment from samples, or they can be developed using an Air-Liquid Interface (ALI) to create a Tumor Microenvironment (TME) model that more closely simulates real-life conditions.

*B. How to co-culture organoids with immune cells*. Co-culturing organoids with immune cells permits the *in vitro* cultivation of organoids under the stimulation of immune cells, thereby creating an environment that more closely resembles *in vivo* growth. There are currently two methods for co-culturing immune cells and organoids: maintaining and expanding the organoid's native immune cells and introducing exogenous immune cells during organoid culture.

*C. How to assess the efficacy of immune checkpoint inhibitors and combine them with chemotherapeutics in the organoid system.* Organoids can foretell how tumors respond to immune checkpoint inhibitors (ICIs), including antibodies against CTLA-4 and PD-1 or PD-L1. PDOs may facilitate the prediction and evaluation of individual tumor responses using PD-1/PD-L1 blockades 124, thereby guiding clinical translational therapy.

*D. How to evaluate the efficacy of adoptive cell therapies such as CAR-T and TILs in in vitro trials* 125, 126. Tumor organoids summarize endogenous antigen expression, can more accurately evaluate the target reactivity, response threshold, and specificity of CAR T cells, can be used for early evaluation of tumor cell specificity, and used as an *in vitro* test platform for optimizing CAR T therapy. As a 3D cell culture model simulating the *in vivo* microenvironment, tumor organoids can form similar spatial structures of organs and differentiate corresponding functions, demonstrating a high degree of tissue consistency and clinical relevance. They can be used as a "patient surrogate" to predict and evaluate the efficacy of TILs, and they can be performed quickly and efficiently, enabling individualized and precise treatment.

***5) Combination therapy optimization*** 127. Organoid DST can aid in the optimization of combination therapies. By testing organoids against various combinations of drugs, clinicians can identify synergistic or additive effects that enhance treatment efficacy. This information helps design personalized combination treatment strategies, potentially improving response rates and overcoming drug resistance. Particularly, organoid DST can be used to optimize existing immunotherapies and combination therapies, as well as to evaluate novel combinations. In the clinic, ICI therapies are increasingly being combined with chemotherapy, radiotherapy, and targeted therapy. Various multicenter, prospective, and large-scale clinical studies of combination therapies are also currently underway, with the outcomes of these clinical trials being validated using organoid models.

**Expert Consensus 7:** Organoid DST can be used to predict the efficacy of chemotherapy, radiotherapy, targeted therapy, and immunotherapy for individual patients.

## 9. What flaws or significant challenges must be solved for the clinical application of organoid DST?

**Figure 4:**
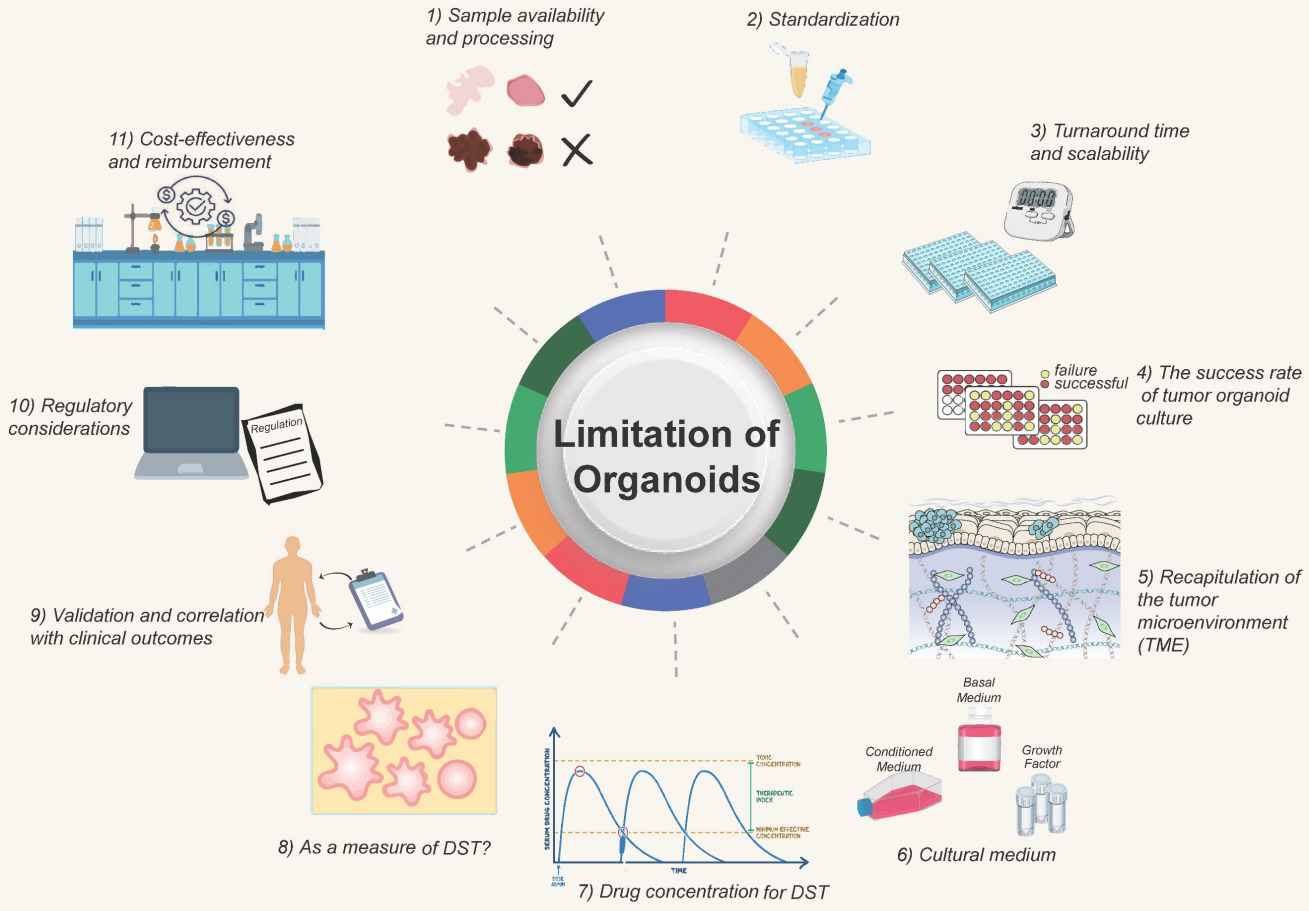
** Challenges and improving direction of organoid models.** The development of organoids currently faces certain challenges, which require researchers to continuously strive for improvement and overcome. They at least include 1) Sample acquisition and processing; 2) Standardized procedures; 3) Turnaround time; 4) Success rate of tumor organoid construction; 5) Simulation of the tumor microenvironment; 6) Optimization of culture media; 7) Determination of drug concentrations; 8) Optimization of tumor organoids; 9) Determination of clinical protocols; 10) Regulatory standards; 11) Consideration of time and financial costs.

The clinical application of DST faces certain challenges and limitations that need to be addressed for broader adoption. Some of the significant challenges and areas that require attention include (Figure 4):

***1) Sample availability and processing***. Obtaining sufficient and high-quality tissue samples for organoid culture can be challenging, especially for certain tumor types or metastatic lesions. Availability of fresh, viable tissue samples is critical for successful organoid establishment. Efforts are needed to optimize sample collection and processing methods, develop strategies for sample preservation, and explore alternative sources such as minimally invasive procedures or liquid biopsies.

***2) Standardization.*** Protocols for organoid culture and DST methodologies need to be standardized to ensure consistent and reproducible results across different laboratories. Standardization efforts should focus on factors such as sample handling, culture conditions, quality control measures, and result interpretation. This is essential for ensuring reliable and comparable outcomes across different institutions and establishing guidelines for clinical implementation.

***3) Turnaround time and scalability.*** Organoid DST should ideally provide rapid results to guide treatment decisions in a clinically relevant timeframe. Efforts are needed to optimize the workflow and reduce the turnaround time for organoid generation and drug sensitivity testing. Additionally, scalability of organoid culture and testing should be addressed to accommodate larger patient populations and facilitate widespread clinical implementation. For patients undergoing postoperative chemotherapy, a drug sensitivity test after 1-3 weeks is acceptable, whereas those getting neoadjuvant chemotherapy or those with advanced tumors require the drug screening to be done as soon as feasible, potentially in less than two weeks. Tumors are heterogeneous, and different cancers or portions of the same tumor develop at varying rates; the organoid DST process will take longer for tumors with a sluggish growth rate. Due to the conflict between time and throughput, it is vital to discuss with clinicians how to choose *qualified* tumor biopsies for various clinical decision-making scenarios 128, 129.

***4) The success rate of tumor organoid culture*.** Organoid culture is affected by tumor cell composition, tumor heterogeneity, cell activity, and so on. It requires that qualified and established process protocols be in place to improve the success rate 130, 131.

***5) Recapitulation of the tumor microenvironment (TME)*.** TME reconstruction is challenging since the tumor may comprise "normal" cells in the TME 132. Current organoid models lack *in vivo* components, such as immune cells 133, endothelial cells 75, and fibroblasts. It is challenging to create organoids composed of vascular and immune cells, but this obstacle should be addressed shortly. When interpreting organoid DST results, the impact of non-tumor cell components on drug testing outcomes should be evaluated 80.

***6) Cultural conditions on drugs*.** The presence of variables that impact signaling pathways, such as the ALK pathway, in the organoid culture medium may affect the natural outcome of testing medications that target this system. Certain cancers require the use of a suitable culture media 134, 135.

***7) Drug concentration for tumor organoid DST*.** Due to pharmacokinetics following administration, the drug concentration administered to patients differs from the that of acting on tumor cells *in vitro*. An excessive drug concentration will lead to cell death, and this cell-killing effect is not caused by the drug's inhibitory effects. For actual clinical application by using PDO-based DST, the optimal drug concentration should be determined by at least two factors. First, serial passaged organoids have been separated from the support and protection of the surrounding TME, every cell in the organoids is a tumor cell at this time. There are two primary methods for determining drug concentrations. The first, and most used, involves calculating the IC50 from dose-response curves 6, 26, 88, 107. The second approach is to refer to the Cmax of a specific drug, as reported in clinical trials or listed on drug labels 136-138. When using gradient concentrations to detect the drug activity on organoids, multiple approaches for detecting total cell activity can be utilized, including live-death staining (calcein-PI staining) 139, organoid size/area quantification 140, MTT 141, MKI67 140, 142, Brud, and CellTiter-ATP detection 143-146. Michael Koch *et al.* examined organoid cell activity after sorafenib treatment by measuring the size/area and MKI67 expression of organoids 140. To obtain the optimal concentration, one must also consider normal organoids in the situation. If it also has a strong inhibitory effect on normal organoids at a particular dosage, it does not accurately reflect the action in patients. Further consideration is needed for the requirements concerning technicians, dispensing balances, and pipettes. The actual drug concentration in organoids will differ from the standard due to human or instrumental error, and batch-to-batch variation will also affect the stability of drug sensitivity.

***8) Whether organoids can reliably be used as a measure of drug sensitivity*.** Due to the various additives in the culture medium, organoids may become contaminated with normal cells during the culturing process. Therefore, quality control measures for organoids are essential before conducting drug sensitivity testing. For instance, NGS sequencing is performed prior to drug sensitivity testing to determine if organoids still contain important mutations related to drug response. If there is a significant deviation from the original tissue, the drug sensitivity results of this strain of organoids should be evaluated with caution. Due to the heterogeneity of tumors, some tumor organoids grew poorly and disintegrated after the addition of drugs, resulting in false positive results. Some tumor organoids develop drug resistance and grow too quickly, resulting in false negative results. Consider repeated inoculation of wells in response to this phenomenon. If the growth of organoids in different wells from the same patient is inconsistent prior to the addition of the drug, this may impact the interpretation of drug sensitivity results. To reduce the variances caused by manual labor, it is possible to use automatic sampling equipment or to drill multiple holes.

***9) Validation and correlation with clinical outcomes*.** Extensive validation studies are required to establish the clinical relevance and predictive accuracy of organoid DST. It is essential to correlate organoid DST results with clinical outcomes to demonstrate its utility in guiding treatment decisions and improving patient outcomes. Longitudinal studies with larger patient cohorts and diverse cancer types are needed to evaluate the clinical value and performance of organoid DST.

***10) Regulatory considerations***. The regulatory landscape for incorporating organoid DST into clinical practice needs to be defined. Regulatory bodies must establish guidelines for validation, quality control, and ethical considerations related to organoid DST. Clear regulations will ensure the safety, reliability, and ethical use of organoid DST in clinical settings.

***11) Cost-effectiveness and reimbursement*.** The cost implications of implementing organoid DST in clinical practice need to be considered. Development of cost-effective protocols, automation of workflows, and optimization of resources are necessary to make organoid DST economically viable. Additionally, reimbursement policies need to be established to support the clinical use of organoid DST and ensure patient access to this promising technology.

**Expert Consensus 8:** Addressing these challenges will require collaborative efforts between researchers, clinicians, regulatory authorities, and healthcare stakeholders to advance the field of organoid DST and enable its widespread clinical application. Timeliness should always be considered when conducting organoid DST. It is possible to produce a report within one to three weeks, and the time required for organoid DST must be progressively shortened through technical development. Continued research, validation studies, technological advancements, and consensus-building efforts are crucial to overcome these limitations and unlock the full potential of organoid DST in precision medicine.

## Supplementary Material

Supplementary Methods and Members of the PDO-based DST Consortium.

## Figures and Tables

**Table 1:** Beneficial outcomes of applying organoid DST in clinical trials

Cancer types	Sample	Therapy	Main Results	Reference
Gastrointestinal cancer	77 organoids from 72 patients	Chemotherapy, Targeted therapy	Organoid DST predicts clinical response: 100% sensitivity, 93% specificity, 88% positive predictive value and 100% negative predictive value	38
Gastrointestinal cancer	11 patients	Chemotherapy, Targeted therapy	Organoid DST predicts clinical response: 82% concordance rate	147
Lung cancer	54 organoids from 36 patients	Chemotherapy, Targeted therapy	Organoid DST predicts clinical response: 84.0% sensitivity, 82.8% specificity	112
Breast cancer	35 patients	Chemotherapy, Targeted therapy, Immunotherapy	Organoid DST predicts clinical response: 82.35% sensitivity, 69.23% specificity, 76.67% accuracy	33
Pancreatic cancer	31 patients	Chemotherapy	Organoid DST divides patients into three groups: sensitive, intermediate, and resistant, with statistical differences in PFS between the groups	148
Pancreatic cancer	16 patients	Chemotherapy	Organoid prediction model allows a successful response prediction in treatment-naïve patients with an accuracy of 91.1% for first-line and 80.0% for second-line regimens, respectively	149
Pancreatic ductal adenocarcinoma	12 organoids	Chemotherapy	A method for classifying PDOs as sensitive or resistant to chemotherapy regimens was developed to predict the clinical outcome of patients.	150
Pancreatic ductal adenocarcinoma	21 organoids	Chemotherapy	Organoid DST responses were not different from patient tumor responses	151

**Table 2:** The difference between conventional fluid-based organoids and PDOs

Differences	Conventional PDOs	Fluid-based organoids
Resources	Surgical or biopsy specimens	Peripheral blood 152, malignant pleural effusion 38, ascites 153, and urine 154
Sample processing	Enzymatic or mechanical dissociation 155	Positive selection immunoaffinity, negative selection immunoaffinity, filtration, label free microfluidic and centrifugation 156
Successful rate	70-80% 38, 157	Peripheral blood 75-90% 157, urine 83% 154, malignant pleural effusion and ascites 40% 158
Strengths	The higher number of tumor cells, the more successful rate	Dual character, non-invasive sampling, can track dynamic change, monitor tumor metastasis, and monitor patients' response to treatment and make timely adjustments 159

## References

[1] Method of the year 2017 (2018). organoids. Nat Methods.

[2] van de Wetering M, Francies HE, Francis JM, Bounova G, Iorio F, Pronk A (2015). Prospective derivation of a living organoid biobank of colorectal cancer patients. Cell.

[3] Yan HHN, Siu HC, Law S, Ho SL, Yue SSK, Tsui WY (2018). A comprehensive human gastric cancer organoid biobank captures tumor subtype heterogeneity and enables therapeutic screening. Cell Stem Cell.

[4] Sachs N, de Ligt J, Kopper O, Gogola E, Bounova G, Weeber F (2018). A living biobank of breast cancer organoids captures disease heterogeneity. Cell.

[5] Beshiri ML, Tice CM, Tran C, Nguyen HM, Sowalsky AG, Agarwal S (2018). A PDX/organoid biobank of advanced prostate cancers captures genomic and phenotypic heterogeneity for disease modeling and therapeutic screening. Clin Cancer Res.

[6] Nuciforo S, Fofana I, Matter MS, Blumer T, Calabrese D, Boldanova T (2018). Organoid models of human liver cancers derived from tumor needle biopsies. Cell Rep.

[7] Beato F, Reverón D, Dezsi KB, Ortiz A, Johnson JO, Chen D (2021). Establishing a living biobank of patient-derived organoids of intraductal papillary mucinous neoplasms of the pancreas. Lab Invest.

[8] Kawasaki K, Toshimitsu K, Matano M, Fujita M, Fujii M, Togasaki K (2020). An organoid biobank of neuroendocrine neoplasms enables genotype-phenotype mapping. Cell.

[9] Jacob F, Salinas RD, Zhang DY, Nguyen PTT, Schnoll JG, Wong SZH (2020). A patient-derived glioblastoma organoid model and biobank recapitulates inter- and intra-tumoral heterogeneity. Cell.

[10] Li YF, Gao Y, Liang BW, Cao XQ, Sun ZJ, Yu JH (2020). Patient-derived organoids of non-small cells lung cancer and their application for drug screening. Neoplasma.

[11] Calandrini C, Schutgens F, Oka R, Margaritis T, Candelli T, Mathijsen L (2020). An organoid biobank for childhood kidney cancers that captures disease and tissue heterogeneity. Nat Commun.

[12] Rumsey JW, Lorance C, Jackson M, Sasserath T, Mcaleer CW, Long CJ (2022). Classical complement pathway inhibition in a "human-on-a-chip" model of autoimmune demyelinating neuropathies. Adv Ther.

[13] Zheng R, Zhang S, Zeng H, Wang S, Sun K, Chen R (2022). Cancer incidence and mortality in China, 2016. J Nat Cancer Cent.

[14] Han B, Zheng R, Zeng H, Wang S, Sun K, Chen R (2024). Cancer incidence and mortality in China, 2022. J Nat Cancer Cent.

[15] Janagama D, Hui SK (2020). 3-D cell culture systems in bone marrow tissue and organoid engineering, and BM phantoms as in vitro models of hematological cancer therapeutics-a review. Materials (Basel).

[16] Lesavage BL, Suhar RA, Broguiere N, Lutolf MP, Heilshorn SC (2022). Next-generation cancer organoids. Nat Mater.

[17] Yuki K, Cheng N, Nakano M, Kuo CJ (2020). Organoid models of tumor immunology. Trends Immunol.

[18] Bose S, Barroso M, Chheda MG, Clevers H, Elez E, Kaochar S (2022). A path to translation: how 3D patient tumor avatars enable next generation precision oncology. Cancer Cell.

[19] Tuveson D, Clevers H (2019). Cancer modeling meets human organoid technology. Science.

[20] Drost J, Clevers H (2018). Organoids in cancer research. Nat Rev Cancer.

[21] Tan R, Zhang Z, Ding P, Liu Y, Liu H, Lu M (2024). A growth factor-reduced culture system for colorectal cancer organoids. Cancer Lett.

[22] Zeng Z, Huang B, Parvez RK, Li Y, Chen J, Vonk AC (2021). Generation of patterned kidney organoids that recapitulate the adult kidney collecting duct system from expandable ureteric bud progenitors. Nat Commun.

[23] Tiriac H, Belleau P, Engle DD, Plenker D, Deschênes A, Somerville TDD (2018). Organoid profiling identifies common responders to chemotherapy in pancreatic cancer. Cancer Dis.

[24] Driehuis E, Kretzschmar K, Clevers H (2020). Establishment of patient-derived cancer organoids for drug-screening applications. Nat Protoc.

[25] Xie X, Li X, Song W (2023). Tumor organoid biobank-new platform for medical research. Sci Rep.

[26] Kim M, Mun H, Sung CO, Cho EJ, Jeon HJ, Chun SM (2019). Patient-derived lung cancer organoids as in vitro cancer models for therapeutic screening. Nat Commun.

[27] Baumann K (2017). Colonic organoids for drug testing and colorectal disease modelling. Nat Rev Mol Cell Biol.

[28] Devarasetty M, Mazzocchi AR, Skardal A (2018). Applications of bioengineered 3D tissue and tumor organoids in drug development and precision medicine: current and future. BioDrugs.

[29] Lo Y, Karlsson K, Kuo CJ (2020). Applications of organoids for cancer biology and precision medicine. Nat Cancer.

[30] Ubink I, Bolhaqueiro ACF, Elias SG, Raats DAE, Constantinides A, Peters NA (2019). Organoids from colorectal peritoneal metastases as a platform for improving hyperthermic intraperitoneal chemotherapy. Br J Surg.

[31] Wang T, Pan W, Zheng H, Zheng H, Wang Z, Li JJ (2021). Accuracy of using a patient-derived tumor organoid culture model to predict the response to chemotherapy regimens in stage IV colorectal cancer: a blinded study. Dis Colon Rectum.

[32] Kim J, Yu D, Kwon Y, Lee KS, Sim SH, Kong SY (2020). Genomic characteristics of triple-negative breast cancer nominate molecular subtypes that predict chemotherapy response. Mol Cancer Res.

[33] Chen P, Zhang X, Ding R, Yang L, Lyu X, Zeng J (2021). Patient-derived organoids can guide personalized-therapies for patients with advanced breast cancer. Adv Sci (Weinh).

[34] Chen JH, Chu XP, Zhang JT, Nie Q, Tang WF, Su J (2020). Genomic characteristics and drug screening among organoids derived from non-small cell lung cancer patients. Thorac Cancer.

[35] Hu Y, Sui X, Song F, Li Y, Li K, Chen Z (2021). Lung cancer organoids analyzed on microwell arrays predict drug responses of patients within a week. Nat Commun.

[36] de Witte CJ, Espejo VJ, Hami N, Lohmussaar K, Kopper O, Vreuls C (2020). Patient-derived ovarian cancer organoids mimic clinical response and exhibit heterogeneous inter- and intrapatient drug responses. Cell Rep.

[37] Mullenders J, de Jongh E, Brousali A, Roosen M, Blom JPA, Begthel H (2019). Mouse and human urothelial cancer organoids: a tool for bladder cancer research. Proc Natl Acad Sci U S A.

[38] Vlachogiannis G, Hedayat S, Vatsiou A, Jamin Y, Fernández-Mateos J, Khan K (2018). Patient-derived organoids model treatment response of metastatic gastrointestinal cancers. Science.

[39] Bian B, Juiz NA, Gayet O, Bigonnet M, Brandone N, Roques J (2019). Pancreatic cancer organoids for determining sensitivity to bromodomain and extra-terminal inhibitors (BETi). Front Oncol.

[40] Artegiani B, Hendriks D, Beumer J, Kok R, Zheng X, Joore I (2020). Fast and efficient generation of knock-in human organoids using homology-independent CRISPR-Cas9 precision genome editing. Nat Cell Biol.

[41] Drost J, van Boxtel R, Blokzijl F, Mizutani T, Sasaki N, Sasselli V (2017). Use of CRISPR-modified human stem cell organoids to study the origin of mutational signatures in cancer. Science.

[42] Artegiani B, van Voorthuijsen L, Lindeboom R, Seinstra D, Heo I, Tapia P (2019). Probing the tumor suppressor function of BAP1 in CRISPR-engineered human liver organoids. Cell Stem Cell.

[43] Kimura M, Iguchi T, Iwasawa K, Dunn A, Thompson WL, Yoneyama Y (2022). En masse organoid phenotyping informs metabolic-associated genetic susceptibility to NASH. Cell.

[44] Schutgens F, Clevers H (2020). Human organoids: tools for understanding biology and treating diseases. Annu Rev Pathol Mech Dis.

[45] Sidhaye J, Knoblich JA (2021). Brain organoids: an ensemble of bioassays to investigate human neurodevelopment and disease. Cell Death Differ.

[46] Streekstra EJ, Russel F, van de Steeg E, de Wildt SN (2021). Application of proteomics to understand maturation of drug metabolizing enzymes and transporters for the optimization of pediatric drug therapy. Drug Discov Today Technol.

[47] Kip AM, Soons Z, Mohren R, Duivenvoorden AAM, Röth AAJ, Cillero-Pastor B (2021). Proteomics analysis of human intestinal organoids during hypoxia and reoxygenation as a model to study ischemia-reperfusion injury. Cell Death Dis.

[48] Xiao Y, Ma D, Yang YS, Yang F, Ding JH, Gong Y (2022). Comprehensive metabolomics expands precision medicine for triple-negative breast cancer. Cell Res.

[49] Hendriks D, Clevers H, Artegiani B (2020). CRISPR-Cas tools and their application in genetic engineering of human stem cells and organoids. Cell Stem Cell.

[50] Bar-Ephraim YE, Kretzschmar K, Clevers H (2020). Organoids in immunological research. Nat Rev Immunol.

[51] Johns AE, Maragakis NJ (2022). Exploring motor neuron diseases using iPSC platforms. Stem Cells.

[52] Wu X, Bos IST, Conlon TM, Ansari M, Verschut V, van der Koog L (2022). A transcriptomics-guided drug target discovery strategy identifies receptor ligands for lung regeneration. Sci Adv.

[53] Ma Y, Yang X, Xin R, Wu T, Shi Y, Dan Zhang D (2021). The power and the promise of organoid models for cancer precision medicine with next-generation functional diagnostics and pharmaceutical exploitation. Transl Oncol.

[54] Tambalo M, Lodato S (2020). Brain organoids: human 3D models to investigate neuronal circuits assembly, function and dysfunction. Brain Res.

[55] Rauth S, Karmakar S, Batra SK, Ponnusamy MP (2021). Recent advances in organoid development and applications in disease modeling. Biochim Biophys Acta Rev Cancer.

[56] Takahashi N, Hoshi H, Higa A, Hiyama G, Tamura H, Ogawa M (2019). An in vitro system for evaluating molecular targeted drugs using lung patient-derived tumor organoids. Cells.

[57] Roy P, Canet-Jourdan C, Annereau M, Zajac O, Gelli M, Broutin S (2017). Organoids as preclinical models to improve intraperitoneal chemotherapy effectiveness for colorectal cancer patients with peritoneal metastases: preclinical models to improve HIPEC. Int J Pharm.

[58] Huang L, Bockorny B, Paul I, Akshinthala D, Frappart P, Gandarilla O (2020). PDX-derived organoids model in vivo drug response and secrete biomarkers. JCI Insight.

[59] Wang B, Gan J, Liu Z, Hui Z, Wei J, Gu X (2022). An organoid library of salivary gland tumors reveals subtype-specific characteristics and biomarkers. J Exp Clin Cancer Res.

[60] Buzzelli JN, Ouaret D, Brown G, Allen PD, Muschel RJ (2018). Colorectal cancer liver metastases organoids retain characteristics of original tumor and acquire chemotherapy resistance. Stem Cell Res.

[61] Armstrong A, Haque MR, Mirbagheri S, Barlass U, Gilbert DZ, Amin J (2021). Multiplex patient-based drug response assay in pancreatic ductal adenocarcinoma. Biomedicines.

[62] Cong B, Thakur T, Uribe AH, Stamou E, Gopinath S, Maddocks O (2023). Colon cancer cells evade drug action by enhancing drug metabolism. bioRxiv.

[63] Tao M, Sun F, Wang J, Wang Y, Zhu H, Chen M (2022). Developing patient-derived organoids to predict PARP inhibitor response and explore resistance overcoming strategies in ovarian cancer. Pharmacol Res.

[64] Nanki Y, Chiyoda T, Hirasawa A, Ookubo A, Itoh M, Ueno M (2020). Patient-derived ovarian cancer organoids capture the genomic profiles of primary tumours applicable for drug sensitivity and resistance testing. Sci Rep.

[65] Han Y, Duan X, Yang L, Nilsson-Payant BE, Wang P, Duan F (2021). Identification of SARS-CoV-2 inhibitors using lung and colonic organoids. Nature.

[66] Herland A, Maoz BM, Das D, Somayaji MR, Prantil-Baun R, Novak R (2020). Quantitative prediction of human pharmacokinetic responses to drugs via fluidically coupled vascularized organ chips. Nat Biomed Eng.

[67] Martin ML, Adileh M, Hsu K, Hua G, Lee SG, Li C (2020). Organoids reveal that inherent radiosensitivity of small and large intestinal stem cells determines organ sensitivity. Cancer Res.

[68] Fu G, Chen S, Liang L, Li X, Tang P, Rao X (2021). SIRT1 inhibitors mitigate radiation-induced GI syndrome by enhancing intestinal-stem-cell survival. Cancer Lett.

[69] Silvia N, Dai G (2020). Cerebral organoids as a model for glioblastoma multiforme. Curr Opin Biomed Eng.

[70] Zhang L, Liu F, Weygant N, Zhang J, Hu P, Qin Z (2021). A novel integrated system using patient-derived glioma cerebral organoids and xenografts for disease modeling and drug screening. Cancer Lett.

[71] Burdis R, Kelly DJ (2021). Biofabrication and bioprinting using cellular aggregates, microtissues and organoids for the engineering of musculoskeletal tissues. Acta Biomater.

[72] Sun L, Wang Y, Cen J, Ma X, Cui L, Qiu Z (2019). Modelling liver cancer initiation with organoids derived from directly reprogrammed human hepatocytes. Nat Cell Biol.

[73] Driehuis E, Spelier S, Beltran HI, de Bree R, M WS, Clevers H (2019). Patient-derived head and neck cancer organoids recapitulate EGFR expression levels of respective tissues and are responsive to EGFR-targeted photodynamic therapy. J Clin Med.

[74] Dayton TL, Alcala N, Moonen L, den Hartigh L, Geurts V, Mangiante L (2023). Druggable growth dependencies and tumor evolution analysis in patient-derived organoids of neuroendocrine neoplasms from multiple body sites. Cancer Cell.

[75] Yu J (2021). Vascularized organoids: a more complete model. Int J Stem Cells.

[76] Scognamiglio G, De Chiara A, Parafioriti A, Armiraglio E, Fazioli F, Gallo M (2019). Patient-derived organoids as a potential model to predict response to PD-1/PD-L1 checkpoint inhibitors. Br J Cancer.

[77] Magre L, Verstegen M, Buschow S, van der Laan L, Peppelenbosch M, Desai J (2023). Emerging organoid-immune co-culture models for cancer research: from oncoimmunology to personalized immunotherapies. J Immunother Cancer.

[78] Verissimo CS, Overmeer RM, Ponsioen B, Drost J, Mertens S, Verlaan-Klink I (2016). Targeting mutant RAS in patient-derived colorectal cancer organoids by combinatorial drug screening. Elife.

[79] Yang S, Ooka M, Margolis RJ, Xia M (2023). Liver three-dimensional cellular models for high-throughput chemical testing. Cell Rep Methods.

[80] Shinozawa T, Kimura M, Cai Y, Saiki N, Yoneyama Y, Ouchi R (2021). High-fidelity drug-induced liver injury screen using human pluripotent stem cell-derived organoids. Gastroenterology.

[81] Brooks A, Liang X, Zhang Y, Zhao C, Roberts MS, Wang H (2021). Liver organoid as a 3D in vitro model for drug validation and toxicity assessment. Pharmacol Res.

[82] Zehir A, Benayed R, Shah RH, Syed A, Middha S, Kim HR (2017). Mutational landscape of metastatic cancer revealed from prospective clinical sequencing of 10,000 patients. Nat Med.

[83] Marquart J, Chen EY, Prasad V (2018). Estimation of the percentage of US patients with cancer who benefit from genome-driven oncology. JAMA Oncol.

[84] Patient-derived organoids predict chemotherapy response Nat Rev Drug Discov. 2019; 18(12): 904.

[85] Organoids may point to best therapy Cancer Dis. 2018; 8(5): 524.

[86] Boj SF, Hwang C, Baker LA, Chio IIC, Engle DD, Corbo V (2015). Organoid models of human and mouse ductal pancreatic cancer. Cell.

[87] Driehuis E, Kolders S, Spelier S, Lõhmussaar K, Willems SM, Devriese LA (2019). Oral mucosal organoids as a potential platform for personalized cancer therapy. Cancer Dis.

[88] Broutier L, Mastrogiovanni G, Verstegen MM, Francies HE, Gavarró LM, Bradshaw CR (2017). Human primary liver cancer-derived organoid cultures for disease modeling and drug screening. Nat Med.

[89] Kopper O, de Witte CJ, Lõhmussaar K, Valle-Inclan JE, Hami N, Kester L (2019). An organoid platform for ovarian cancer captures intra- and interpatient heterogeneity. Nat Med.

[90] Jansen J, Reimer KC, Nagai JS, Varghese FS, Overheul GJ, de Beer M (2022). SARS-CoV-2 infects the human kidney and drives fibrosis in kidney organoids. Cell Stem Cell.

[91] Karkampouna S, La Manna F, Benjak A, Kiener M, De Menna M, Zoni E (2021). Patient-derived xenografts and organoids model therapy response in prostate cancer. Nat Commun.

[92] Jin M, Han R, Qiu G, Ju X, Lou G, Jin W (2018). Organoids: an intermediate modeling platform in precision oncology. Cancer Lett.

[93] Abdel FA, Daza B, Rustandi G, Berrocal-Rubio MA, Gorissen B, Poovathingal S (2021). Actuation enhances patterning in human neural tube organoids. Nat Commun.

[94] Johnson KA, Destefanis RA, Emmerich PB, Grogan PT, Kratz JD, Makkar SK (2020). Human colon organoids and other laboratory strategies to enhance patient treatment selection. Curr Treat Options Oncol.

[95] Lee SH, Hu W, Matulay JT, Silva MV, Owczarek TB, Kim K (2018). Tumor evolution and drug response in patient-derived organoid models of bladder cancer. Cell.

[96] Wang T, Song W, Meng Q, Qu C, Guo S, Wang Y (2024). Tumorigenicity and prediction of clinical prognosis of patient-derived gastric cancer organoids. Clin Transl Med.

[97] Ganesh K, Wu C, O Rourke KP, Szeglin BC, Zheng Y, Sauvé CG (2019). A rectal cancer organoid platform to study individual responses to chemoradiation. Nat Med.

[98] Fatehullah A, Tan SH, Barker N (2016). Organoids as an in vitro model of human development and disease. Nat Cell Biol.

[99] Choo N, Ramm S, Luu J, Winter JM, Selth LA, Dwyer AR (2021). High-throughput imaging assay for drug screening of 3D prostate cancer organoids. SLAS Discov.

[100] Hofer M, Lutolf MP (2021). Engineering organoids. Nat Rev Mater.

[101] Choudhury D, Ashok A, Naing MW (2020). Commercialization of organoids. Trends Mol Med.

[102] Seppala TT, Zimmerman JW, Suri R, Zlomke H, Ivey GD, Szabolcs A (2022). Precision medicine in pancreatic cancer: patient-derived organoid pharmacotyping is a predictive biomarker of clinical treatment response. Clin Cancer Res.

[103] Yao Y, Xu X, Yang L, Zhu J, Wan J, Shen L (2020). Patient-derived organoids predict chemoradiation responses of locally advanced rectal cancer. Cell Stem Cell.

[104] Linkous A, Balamatsias D, Snuderl M, Edwards L, Miyaguchi K, Milner T (2019). Modeling patient-derived glioblastoma with cerebral organoids. Cell Rep.

[105] Yamazaki S, Ohka F, Hirano M, Shiraki Y, Motomura K, Tanahashi K (2021). Newly established patient-derived organoid model of intracranial meningioma. Neuro Oncol.

[106] Farshadi EA, Chang J, Sampadi B, Doukas M, Van T LF, van der Sijde F (2021). Organoids derived from neoadjuvant FOLFIRINOX patients recapitulate therapy resistance in pancreatic ductal adenocarcinoma. Clin Cancer Res.

[107] Demyan L, Habowski AN, Plenker D, King DA, Standring OJ, Tsang C (2022). Pancreatic cancer patient-derived organoids can predict response to neoadjuvant chemotherapy. Ann Surg.

[108] Guo S, Shen J, Gao J, Shi X, Gao S, Wang H (2020). A preoperative risk model for early recurrence after radical resection may facilitate initial treatment decisions concerning the use of neoadjuvant therapy for patients with pancreatic ductal adenocarcinoma. Surgery.

[109] Calandrini C, van Hooff SR, Paassen I, Ayyildiz D, Derakhshan S, Dolman M (2021). Organoid-based drug screening reveals neddylation as therapeutic target for malignant rhabdoid tumors. Cell Rep.

[110] Yoon AJ, Santella RM, Wang S, Kutler DI, Carvajal RD, Philipone E (2021). MicroRNA-based cancer mortality risk scoring system and hTERT expression in early-stage oral squamous cell carcinoma. J Oncol.

[111] Vives J, Batlle-Morera L (2020). The challenge of developing human 3D organoids into medicines. Stem Cell Res Ther.

[112] Wang H, Zhang C, Peng K, Chen Z, Su J, Li Y (2023). Using patient-derived organoids to predict locally advanced or metastatic lung cancer tumor response: a real-world study. Cell Rep Med.

[113] Nicolas AM, Pesic M, Engel E, Ziegler PK, Diefenhardt M, Kennel KB (2022). Inflammatory fibroblasts mediate resistance to neoadjuvant therapy in rectal cancer. Cancer Cell.

[114] Hubert CG, Rivera M, Spangler LC, Wu Q, Mack SC, Prager BC (2016). A three-dimensional organoid culture system derived from human glioblastomas recapitulates the hypoxic gradients and cancer stem cell heterogeneity of tumors foundin vivo. Cancer Res.

[115] WHO (2012). Surveillance guidelines for measles, rubella and congenital rubella syndrome in the WHO European region. World Health Organization: Geneva.

[116] Shi R, Radulovich N, Ng C, Liu N, Notsuda H, Cabanero M (2020). Organoid cultures as preclinical models of non-small cell lung cancer. Clin Cancer Res.

[117] Li J, Chen Y, Zhang Y, Peng X, Wu M, Chen L (2023). Clinical value and influencing factors of establishing stomach cancer organoids by endoscopic biopsy. J Cancer Res Clin Oncol.

[118] Tsai KK, Huang SS, Northey JJ, Liao WY, Hsu CC, Cheng LH (2022). Screening of organoids derived from patients with breast cancer implicates the repressor NCOR2 in cytotoxic stress response and antitumor immunity. Nat Cancer.

[119] Zhang Z, Shiratsuchi H, Palanisamy N, Nagrath S, Ramnath N (2017). Expanded circulating tumor cells from a patient with alk-positive lung cancer present with eml4-alk rearrangement along with resistance mutation and enable drug sensitivity testing: a case study. J Thorac Oncol.

[120] Szebényi K, Wenger LMD, Sun Y, Dunn AWE, Limegrover CA, Gibbons GM (2021). Human ALS/FTD brain organoid slice cultures display distinct early astrocyte and targetable neuronal pathology. Nat Neurosci.

[121] Tang F, Xu D, Wang S, Wong CK, Martinez-Fundichely A, Lee CJ (2022). Chromatin profiles classify castration-resistant prostate cancers suggesting therapeutic targets. Science.

[122] Holokai L, Chakrabarti J, Lundy J, Croagh D, Adhikary P, Richards SS (2020). Murine- and human-derived autologous organoid/immune cell co-cultures as pre-clinical models of pancreatic ductal adenocarcinoma. Cancers (Basel).

[123] Chakrabarti J, Koh V, So J, Yong WP, Zavros Y (2021). A preclinical human-derived autologous gastric cancer organoid/immune cell co-culture model to predict the efficacy of targeted therapies. J Vis Exp.

[124] Cimen Bozkus C, Bhardwaj N (2021). Tumor organoid-originated biomarkers predict immune response to PD-1 blockade. Cancer Cell.

[125] Schnalzger TE, de Groot MH, Zhang C, Mosa MH, Michels BE, Röder J (2019). 3D model for CAR-mediated cytotoxicity using patient-derived colorectal cancer organoids. EMBO J.

[126] Xu H, Lyu X, Yi M, Zhao W, Song Y, Wu K (2018). Organoid technology and applications in cancer research. J Hematol Oncol.

[127] Jain HV, Sorribes IC, Handelman SK, Barnaby J, Jackson TL (2021). Standing variations modeling captures inter-individual heterogeneity in a deterministic model of prostate cancer response to combination therapy. Cancers (Basel).

[128] Veninga V, Voest EE (2021). Tumor organoids: opportunities and challenges to guide precision medicine. Cancer Cell.

[129] Rossi G, Manfrin A, Lutolf MP (2018). Progress and potential in organoid research. Nat Rev Genet.

[130] Garreta E, Kamm RD, Chuva De Sousa Lopes SM, Lancaster MA, Weiss R, Trepat X (2021). Rethinking organoid technology through bioengineering. Nat Mater.

[131] Bleijs M, van de Wetering M, Clevers H, Drost J (2019). Xenograft and organoid model systems in cancer research. EMBO J.

[132] Saglam-Metiner P, Gulce-Iz S, Biray-Avci C (2019). Bioengineering-inspired three-dimensional culture systems: organoids to create tumor microenvironment. Gene.

[133] Neal JT, Li X, Zhu J, Giangarra V, Grzeskowiak CL, Ju J (2018). Organoid modeling of the tumor immune microenvironment. Cell.

[134] Nishinakamura R (2019). Human kidney organoids: progress and remaining challenges. Nat Rev Nephrol.

[135] Marsee A, Roos F, Verstegen M, Gehart H, de Koning E, Lemaigre F (2021). Building consensus on definition and nomenclature of hepatic, pancreatic, and biliary organoids. Cell Stem Cell.

[136] Wu Y, Hung Y, Chiu N, Lee R, Li C, Chao Y (2022). Correlation between drug sensitivity profiles of circulating tumour cell-derived organoids and clinical treatment response in patients with pancreatic ductal adenocarcinoma. Eur J Cancer.

[137] Ooft SN, Weeber F, Schipper L, Dijkstra KK, Mclean CM, Kaing S (2021). Prospective experimental treatment of colorectal cancer patients based on organoid drug responses. ESMO Open.

[138] Narasimhan V, Wright JA, Churchill M, Wang T, Rosati R, Lannagan TRM (2020). Medium-throughput drug screening of patient-derived organoids from colorectal peritoneal metastases to direct personalized therapy. Clin Cancer Res.

[139] Mukundan S, Bell J, Teryek M, Hernandez C, Love AC, Parekkadan B (2022). Automated assessment of cancer drug efficacy on breast tumor spheroids in Aggrewell™400 plates using image cytometry. J Fluoresc.

[140] Koch M, Nickel S, Lieshout R, Lissek SM, Leskova M, van der Laan LJW (2022). Label-free imaging analysis of patient-derived cholangiocarcinoma organoids after sorafenib treatment. Cells.

[141] Iwama T, Fujiya M, Konishi H, Tanaka H, Murakami Y, Kunogi T (2021). Bacteria-derived ferrichrome inhibits tumor progression in sporadic colorectal neoplasms and colitis-associated cancer. Cancer Cell Int.

[142] Pennel K, Hatthakarnkul P, Wood CS, Lian GY, Al-Badran S, Quinn JA (2024). JAK/STAT3 represents a therapeutic target for colorectal cancer patients with stromal-rich tumors. J Exp Clin Cancer Res.

[143] Sendi H, Mead I, Wan M, Mehrab-Mohseni M, Koch K, Atala A (2018). miR-122 inhibition in a human liver organoid model leads to liver inflammation, necrosis, steatofibrosis and dysregulated insulin signaling. PLoS One.

[144] Song X, Hou K, Zhou H, Yang J, Cao T, Zhang J (2024). Liver organoids and their application in liver cancer research. Regen Ther.

[145] Li L, Knutsdottir H, Hui K, Weiss MJ, He J, Philosophe B (2019). Human primary liver cancer organoids reveal intratumor and interpatient drug response heterogeneity. JCI Insight.

[146] Lee J, Lee S, Lee S, Choi J, Lim S, Kim M (2023). Antiproliferative activity of krukovine by regulating transmembrane protein 139 (TMEM139) in oxaliplatin-resistant pancreatic cancer cells. Cancers (Basel).

[147] Hogenson TL, Xie H, Phillips WJ, Toruner MD, Li JJ, Horn IP (2022). Culture media composition influences patient-derived organoid ability to predict therapeutic responses in gastrointestinal cancers. JCI Insight.

[148] Shi X, Li Y, Yuan Q, Tang S, Guo S, Zhang Y (2022). Integrated profiling of human pancreatic cancer organoids reveals chromatin accessibility features associated with drug sensitivity. Nat Commun.

[149] Beutel AK, Schütte L, Scheible J, Roger E, Müller M, Perkhofer L (2021). A prospective feasibility trial to challenge patient-derived pancreatic cancer organoids in predicting treatment response. Cancers (Basel).

[150] Grossman JE, Muthuswamy L, Huang L, Akshinthala D, Perea S, Gonzalez RS (2022). Organoid sensitivity correlates with therapeutic response in patients with pancreatic cancer. Clin Cancer Res.

[151] Kang YA, Deng J, Ling J, Li X, Chiang Y, Koay EJ (2022). 3D imaging analysis on an organoid-based platform guides personalized treatment in pancreatic ductal adenocarcinoma. J Clin Invest.

[152] Gao D, Vela I, Sboner A, Iaquinta PJ, Karthaus WR, Gopalan A (2014). Organoid cultures derived from patients with advanced prostate cancer. Cell.

[153] Li J, Xu H, Zhang L, Song L, Feng D, Peng X (2019). Malignant ascites-derived organoid (MADO) cultures for gastric cancer in vitro modelling and drug screening. J Cancer Res Clin Oncol.

[154] Walz S, Pollehne P, Geng R, Schneider J, Maas M, Aicher WK (2023). A protocol for organoids from the urine of bladder cancer patients. Cells.

[155] Zhou C, Wu Y, Wang Z, Liu Y, Yu J, Wang W (2023). Standardization of organoid culture in cancer research. Cancer Med.

[156] Rupp B, Ball H, Wuchu F, Nagrath D, Nagrath S (2022). Circulating tumor cells in precision medicine: challenges and opportunities. Trends Pharmacol Sci.

[157] Yang H, Cheng J, Zhuang H, Xu H, Wang Y, Zhang T (2024). Pharmacogenomic profiling of intra-tumor heterogeneity using a large organoid biobank of liver cancer. Cancer Cell.

[158] Choi W, Kim Y, Woo SM, Yu Y, Lee MR, Lee WJ (2023). Establishment of patient-derived organoids using ascitic or pleural fluid from cancer patients. Cancer Res Treat.

[159] Huang L, Xu Y, Wang N, Yi K, Xi X, Si H (2024). Next-generation preclinical functional testing models in cancer precision medicine: CTC-derived organoids. Small Methods.

