# Supplementary material for: Building consensus on the application of organoid-based drug sensitivity testing in cancer precision medicine and drug development
Source: Theranostics. 2024 May 27;14(8):3300–16. doi: 10.7150/thno.96027 (PMC11155402; doi:10.7150/thno.96027)
Supplement: Supplementary file 1 — Supplementary Methods and Members of the PDO-based DST Consortium. [file thnov14p3300s1.pdf]

Supplementary Information

Supplementary Methods

Expert Invitation

Scientists, henceforth referred to as experts, were invited based on their expertise in clinical and academic oncology (solid malignancies). Invitations were extended to individuals who had made significant contributions (either as first, second, or senior authors) in peer-reviewed publications focusing on cancer organoids. Upon invitation, experts could nominate additional individuals, subject to evaluation by the core team (DX, AH, RZ, LL, YC, SG, and YL). Following the initial review process, no further invitations were extended.

Consensus Building

To achieve consensus, a questionnaire was developed and reviewed by the core team before distribution to invited experts. Consensus was defined as ⩾90% agreement on a single question. Experts were allowed to propose additional responses during initial and subsequent discussions. Open discussions were conducted via Tecent virtual meetings to deliberate on consensus statements. After each meeting, a summary document of responses was circulated to participants, along with an invitation to complete a revised version. Final propositions were formulated by the core team based on these discussions. All experts were given time to review and express their views. Ultimately, every expert who completed the questionnaires concurred with the outcomes and contributed to the consensus process.

Members of the PDO-based DST Consortium

Dongxi Xiang[1,2], Aina He[3], Rong Zhou[4-5], Yonggang Wang[3], Xiuying Xiao[6], Ting Gong[7], Wenyan Kang[8-9], Xiaolin Lin[6#], Xiaochen Wang[10], Yue He[4-5], Jian Zhang[11-12], Jianjun Zhang[13], Jing Sun[14], Ligang Xing[15], Changchun Zhou[16], Zengjun Liu[17], Cheng Yang[18], Qingcheng Yang[19], Zhan Wang[20], Bin Wang[21-22], Lidong Wang[23], Zebing Liu[24], Fei Jiang[25], Hongqi Chen[26], Yuehong Cui[27-28], Tianshu Liu[27-28], Yalong Wang[29], Junhan Zhao[30-31], David T. Breault[32], Nima Saeidi[33], Chuxia Deng[34-35], Wei Duan[36], Jiabei Wang[37-38], Ganglong Gao[2], Gengming Niu[39], Laiping Zhong[40], Xiaozhe Qian[41], Ai Zhuang[42], Bin Jia[43], Jin Li[44], Ying Xie[45], Xiaonan Kang[46], Guiying Wei[39], Shengping Xiao[39], Jianming Zhang[47], Zhihui Li[48], Jiaye Liu[48], Xianming Kong[49], Shuqian Xia[50], Yuanbo Wu[51], Maorong Chen[52], Jie Cao[53], Xu'an Wang[2], Shuai Gong[54], Organoid Committee of Shanghai Association for Promotion of Healthy Life[55]

[1] State Key Laboratory of Systems Medicine for Cancer, Shanghai Cancer Institute, Shanghai Jiaotong University School of Medicine, Shanghai 200232, PRC
[2] Department of Biliary-Pancreatic Surgery, Renji Hospital Affiliated to Shanghai Jiaotong University School of Medicine, Shanghai 200127, PRC
[3] Department of Oncology, Shanghai Jiaotong University Affiliated Sixth People's Hospital, Shanghai 200233 PRC
[4] Department of Oral and Maxillofacial-Head and Neck Oncology, Ninth People's Hospital, Shanghai Jiaotong University School of Medicine, Shanghai 200125, PRC
[5] National Center of Stomatology, National Clinical Research Center for Oral Disease, Shanghai 200011, PRC
[6] Department of Oncology, Ren Ji Hospital, Shanghai Jiaotong University School of Medicine, Shanghai 200127, PRC
[7] Department of Oncology, Tianjin Medical University General Hospital, Tianjin 300052, PRC
[8] Department of Neurology and Institute of Neurology, Ruijin Hospital Affiliated to Shanghai Jiaotong University School of Medicine, Shanghai 200025, PRC
[9] Department of Neurology, Ruijin Hospital Affiliated to Shanghai Jiaotong University School of Medicine (Boao Research Hospital), Hainan 571434, PRC
[10] Department of Surgical Oncology, Second Affiliated Hospital, Zhejiang University School of Medicine, No. 88, Jiefang Road, Hangzhou, Zhejiang 310009, PRC
[11] Department of Phase I Clinical Trial Center, Fudan University Shanghai Cancer Center, No. 270, Dong'an Road, Shanghai 200032, PRC
[12] Department of Medical Oncology, Fudan University Shanghai Cancer Center, Shanghai 200032, PRC
[13] Department of Oncology, Shanghai Jiaotong University Affiliated Tongren Hospital, Shanghai 200127, PRC
[14] Department of General Surgery, Ruijin Hospital, Shanghai Jiaotong University School of Medicine, 197 Ruijin Er Road, Shanghai 200127, PRC
[15] Department of Radiation Oncology, Shandong Cancer Hospital and Institute, Shandong First Medical University, Shandong Academy of Medical Science, Jinan 250117, Shandong, PRC
[16] Biobank, Cancer Research Center, Shandong Cancer Hospital and Institute, Shandong First Medical University, Shandong Academy of Medical Sciences, Jinan, Shandong 250117, PRC
[17] Rare Tumors Department, Shandong Cancer Hospital and Institute, Shandong First Medical University and Shandong Academy of Medical Sciences, 440 Jiyan Road, Jinan, Shandong 250117, PRC
[18] Department of Orthopedic Oncology, Changzheng Hospital, Second Military Medical University, 415 Fengyang Road, Shanghai 200003, PRC
[19] Department of Orthopedics, Shanghai Jiaotong University Affiliated Sixth People's Hospital, No. 600 Yishan Road, Shanghai 200233, PRC
[20] Department of Medical Oncology, Changzheng Hospital, Second Military Medical University, Shanghai 200003, PRC
[21] Institute of Pathology and Southwest Cancer Center, and Key Laboratory of Tumor Immunopathology of Ministry of Education of China, Southwest Hospital, Army Medical University (Third Military Medical University), Chongqing 400042, PRC
[22] Department of Gastroenterology, Daping Hospital, Army Medical University (Third Military Medical University), Chongqing 400042, PRC
[23] State Key Laboratory of Esophageal Cancer Prevention and Treatment and Henan Key Laboratory for Esophageal Cancer Research of The First Affiliated Hospital, Zhengzhou University, Zhengzhou, Henan 450052, PRC
[24] Department of Pathology, Shanghai Jiao Tong University School of Medicine, Shanghai, China 200232, PRC

[25] Department of Gastroenterology, Changhai Hospital of Second Military Medical University, 168 Changhai Road, Shanghai 200433, PRC
[26] Department of General Surgery, Shanghai Jiaotong University Affiliated Sixth People's Hospital, Shanghai 200233, PRC
[27] Department of Medical Oncology, Zhongshan Hospital, Fudan University, Shanghai 200032, PRC
[28] Cancer Center, Zhongshan Hospital, Fudan University, Shanghai 200032, PRC
[29] Guangzhou National Laboratory, Guangzhou 510005, PRC
[30] Department of Biostatistics, Harvard T. H. Chan School of Public Health, Boston, Massachusetts, MA 02115, USA
[31] Department of Biomedical Informatics, Harvard Medical School, Boston, Massachusetts, MA 02115, USA
[32] Department of Pediatrics, Boston Children's Hospital, Harvard Medical School, 300 Longwood Avenue, Boston, MA 02115, USA
[33] Center for Engineering in Medicine & Surgery, MGH, 51 Blossom St., Boston, MA 02114, USA
[34] Cancer Center, Faculty of Health Sciences, University of Macau, Macau SAR, 999078, PRC
[35] MOE Frontier Science Centre for Precision Oncology, Cancer Center, Faculty of Health Sciences, University of Macau, Taipa, Macau SAR 999078, PRC
[36] School of Medicine and Centre for Molecular and Medical Research, School of Medicine, Deakin University, 75 Pigdons Road, Waurn Ponds, Victoria 3216, Australia.
[37] Department of Hepatobiliary Surgery, The First Affiliated Hospital of USTC, Division of Life Sciences and Medicine, University of Science and Technology of China, Anhui 230001, PRC
[38] Anhui Province Key Laboratory of Hepatopancreatobiliary Surgery, Anhui Provincial Clinical Research Center for Hepatobiliary Diseases, Hefei, Anhui 230001, PRC
[39] Shanghai OneTar Biomedicine, Shanghai 201203, PRC
[40] Department of Stomatology, Oralmaxillofacial Head and Neck Surgery, Huashan Hospital, Fudan University, Shanghai 200040, PRC
[41] Department of Thoracic Surgery, Renji Hospital, School of Medicine, Shanghai Jiaotong University, Shanghai 200127, PRC
[42] Department of Ophthalmology, Ninth People's Hospital, Shanghai Jiaotong University School of Medicine, Shanghai 200125, PRC
[43] Lung Cancer Department, Tianjin Cancer Hospital, Tianjin 300060, PRC
[44] Department of Gynecologic Oncology, Cancer Hospital of Fudan University, Shanghai 200032, PRC
[45] Life Sciences Institute, Guangxi Medical University, Nanning, Guangxi, 530021, PRC
[46] Department of Biobank, Renji Hospital, Shanghai Jiaotong University School of Medicine, Shanghai 200127, PRC
[47] Institute of Translational Medicine, Zhangjiang Institute for Advanced Study, Shanghai Jiao Tong University, Shanghai 200240, PRC
[48] Department of Thyroid and Parathyroid Surgery West China Hospital, Sichuan University Chengdu 610041, PRC
[49] Shanghai Institute of Health Sciences, Shanghai 201318, PRC
[50] Department of Otolaryngology, Yangxin County People's Hospital, Hubei 435200, PRC
[51] Department of Ultrasound, Yangxin County People's Hospital, Hubei 435200, PRC
[52] Center for Life Science, Yunnan Key Laboratory of Cell Metabolism and Diseases, School of Life Sciences, Yunnan University, Kunming 650500, PRC
[53] Department of Hepatology Surgery, Renji Hospital Affiliated to Shanghai Jiaotong University School of Medicine, Shanghai 200127, PRC
[54] Division of Gastroenterology and Hepatology, Renji Hospital Affiliated to Shanghai Jiaotong University School of Medicine, Shanghai 200127, PRC
[55] NO. 57 Huanlong Road, Shanghai 200127, PRC
